# Anti-Migratory and Pro-Apoptotic Properties of Parvifloron D on Triple-Negative Breast Cancer Cells

**DOI:** 10.3390/biom10010158

**Published:** 2020-01-19

**Authors:** Nuno Saraiva, João G. Costa, Catarina Reis, Nuno Almeida, Patrícia Rijo, Ana Sofia Fernandes

**Affiliations:** 1 CBIOS, Universidade Lusófona Research Center for Biosciences & Health Technologies, Campo Grande 376, 1749-024 Lisboa, Portugal; nuno.saraiva@ulusofona.pt (N.S.); jgcosta@ulusofona.pt (J.G.C.); catarinareis@ff.ulisboa.pt (C.R.); nunoallmeida@live.com (N.A.); patricia.rijo@ulusofona.pt (P.R.); 2 Research Institute for Medicines (iMed.ULisboa), Faculty of Pharmacy, Universidade de Lisboa, Av. Professor Gama Pinto, 1649-003 Lisboa, Portugal

**Keywords:** natural compounds, breast cancer, cell viability, cell migration, apoptosis

## Abstract

Medicinal plants are important sources of new bioactive compounds with potential anticancer activity. Parvifloron D (ParvD) is an abietane diterpenoid, isolated in high amounts from *Plectranthus ecklonii* Benth. Previous reports have suggested potential therapeutic properties for ParvD. ParvD has shown pro-apoptotic and cytotoxic effects in leukemia and melanoma cell lines. However, to the best of our knowledge, there are no studies in triple-negative breast cancer (TNBC) models. TNBC is a breast cancer subtype characterized by an aggressive behavior with poor clinical outcomes and weak overall therapeutic responses to the current treatment options. This work aimed at evaluating the anticancer effect of ParvD in MDA-MB-231 cells, a model of human TNBC. To obtain sufficient amounts of purified ParvD the efficiency of several extraction methods was compared. ParvD (0.1–10 µM) decreased cell viability in a concentration-dependent manner. Treatment with ParvD (5 µM) significantly increased the percentage of apoptotic nuclei and exposure to 3 µM ParvD increased the sub-G1 population. Since altered cell adherence, migration, and invasion are determinant processes for the formation of metastases, the effect of ParvD on these cellular processes was tested. Although treatment with ParvD (1 µM) had no effect on cell-substrate attachment, ParvD (1 µM) significantly reduced cell chemotaxis and invasion. This is the first report describing the proapoptotic effect of ParvD in TNBC cells. Moreover, for the first time we have shown that ParvD reduces cell motility, unraveling potential anti-metastatic properties.

## 1. Introduction

Phytotherapy is as old as the human civilization itself and has been used over the centuries for a variety of health conditions [1,2]. Nowadays, multiple efforts are in motion to assess the potential pharmacological activity of plant-derived compounds as antimicrobial, antifungal, anti-inflammatory, antioxidant, and anticancer agents [2,3]. Many existing classical anticancer drugs have a plant source. This is the case for taxol (derived from *Taxus brevifolia*), camptothecin (derived from *Camptotheca acuminate*), among others [1,4]. Plants constitute thus a prolific source to search for new drugs for cancer prevention or treatment [1]. The development of extraction and synthesis techniques aims to allow the mass production of anticancer bioactive plant derivatives [4].

**Figure 1:**
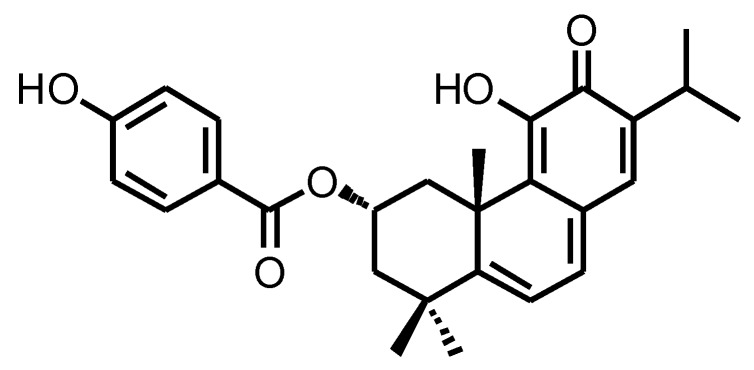
Chemical structure of Parvifloron D.

Plants from the *Plectranthus* genus, which belongs to the family Lamiaceae have been studied for their multiple pharmacological properties [5,6]. *Plectranthus* species are rich in naturally occurring compounds like the abietane diterpenes, which exhibit a broad spectrum of biological activities, including anticancer activity [5,7,8,9,10]. Parvifloron D (ParvD; Figure 1) is a major diterpenoid of *P. ecklonii* and its anticancer properties were described against human leukemia, melanoma, colon, pancreatic, and non-metastatic breast cancer cell lines, as well in human keratinocytes [7,11,12,13].

Breast cancer is the most prevalent type of cancer in women from developed countries, accounting for approximately 20% of female cancer deaths. Breast cancer has a complex and heterogeneous pattern regarding histology, mutations, metastatic potential, cellular origin, progression, treatment response, and clinical outcome [14]. Triple-negative breast cancer (TNBC) is characterized by the lacking expression of the three markers that define breast cancer subtypes and treatment, namely, the estrogen receptor, progesterone receptor, and human epidermal growth factor receptor 2 (HER-2). TNBC represents approximately 20% of all breast cancers [15,16,17]. TNBC has generally a more aggressive behavior and is associated with a poor clinical outcome [16,17,18]. The treatment guidelines for TNBC are currently limited to systemic chemotherapy for early and later stages, with unsatisfactory results, shown by weak overall therapeutic response rates and low median progression-free survival [16,17,19]. The aim of this study was to isolate ParvD from *P. ecklonii* Benth by a selected extraction method and to assess the impact of this isolated compound on TNBC cell motility, cell cycle and cell death using an in vitro model of TNBC. Here, we describe for the first time the proapoptotic and anti-migratory effects of ParvD in a TNBC cell model.

## 2. Materials and Methods

### 2.1. Chemicals

Dulbecco’s modified Eagle’s medium (DMEM), fetal bovine serum (FBS), penicillin-streptomycin solution, trypsin, and crystal violet (CV) were purchased from Sigma-Aldrich (St Louis, MO, USA). Dimethylsulphoxide (DMSO), ethanol, and acetic acid were purchased from Merck (Darmstadt, Germany). Matrigel was purchased from BD Biosciences (San Jose, CA, USA). DAPI, PI, and RNAase were purchased from Thermo Fisher Scientific (Waltham, MA, USA).

*P. ecklonii* Benth air-dried and powdered plant material was given by the Faculty of Pharmacy of the University of Lisbon and it was collected from seeds provided by the herbarium of the National Botanical Garden of Kirstenbosch, South Africa. Voucher specimens (S/No. LISC) have been deposited in the herbarium of the Tropical Research Institute in Lisbon [11]. Acetone and other organic solvents were from analytic grade and provided by (VWR international S.A.S., Briare, France); Silica for the isolation was obtained from Merck (grade 60, 230–400 mesh, Merck KGaA, Darmstadt, Germany).

#### *P. ecklonii* Extracts Preparation

In this study, different extracts of *P. ecklonii* were prepared using multiple extraction techniques (decoction, infusion, microwave, supercritical fluid, and ultrasound-assisted extractions) [9]. Acetone, water, and supercritical CO_2_ were used as extraction solvents for the different extraction procedures. The extraction quantification of ParvD was assessed by HPLC-DAD. Aqueous and acetonic extracts were prepared using 10% (w of plant, g; v of extraction solvent, mL).

Aqueous plant extracts were prepared by:

1. Infusion, using freshly boiled distilled water.

2. Decoction, boiling distilled water with plant material for 10 min.

3. Microwave extraction, using distilled water under a conventional microwave for 2 min at continuous irradiation of 2.45 GHz.

4. Ultrasound extraction using distilled water in an ultrasonic bath (Sonorex Super RK 510 H; Bandelin, Berlin, Germany) at room temperature for 30 min.

All aqueous extracts were further filtered using a Whatman paper no 5 (Whatman, Inc., Clifton, NJ, USA) and lyophilized (Freezone 2.5 L, Freeze-dryer Labconco, Kansas City, MO, USA) prior to use in HPLC analysis [9].

Acetonic extracts were obtained using an ultrasonic bath (Sonorex Super RK 510 H; Bandelin, Berlin, Germany) operated for 1 h, at 35 Hz with maximum input power of 320 W in ultrasound extraction or maceration 1 h under magnetic stirring.

The obtained organic extracts were filtered, and the solvent was removed by rotary evaporation [9].

The supercritical fluid extraction (SFE) was done as previously described [20].

### 2.2. Isolation and Quantification of ParvD by HPLC-DAD

An authentic sample of ParvD was isolated from an acetonic ultrasound-assisted extract of *P. ecklonii* Benth., as described in a previous work [11]. The authentic sample of ParvD was in agreement with the spectroscopic means (purity assessed by 1H-NMR spectrum—Figure S1. see Supporting Information) and was further used in the HPLC-DAD quantification and biological assays. The HPLC analysis was performed in a high-performance liquid chromatography with diode array detection (HPLC-DAD) with an Agilent Technologies 1200 Infinity Series LC system (Agilent Technologies, Santa Clara, CA, USA) coupled to a diode array detector (DAD), using a ChemStation Software and a reverse phase LiChrospher^®^ 100 RP-18 5 μm (4.0 × 250 mm) column (Merck). The ParvD of all the extracts was quantified by injecting 20 µL of each sample at 1 mg/mL, using a gradient composed of Solution A (methanol), Solution B (acetonitrile), and Solution D (0.3% trichloroacetic acid in water) as follows: 0 min, 15% A, 5% B, and 80% D; 20 min, 80% A, 10% B, and 10% D; 25 min, 80% A, 10% B, and 10% D. The flow rate was set at 1 mL/min. ParvD was run under the same conditions in methanol, and the detection was carried out between 200 and 600 nm with a diode array detector (DAD). All analyses were performed in triplicate.

### 2.3. Cell Culture

The human breast cancer cell line MDA-MB-231 was obtained from ATCC (HTB-26). Cells were cultured in DMEM supplemented with 10% fetal bovine serum, 100 U/mL penicillin, and 0.1 mg/mL streptomycin. The cultures were maintained at 37 °C, under a humidified atmosphere containing 5% CO_2_ in the air [21].

### 2.4. Cell Viability

Cell viability was evaluated by the crystal violet (CV) staining assay. Approximately 5 × 10^3^ cells in 200 μL of culture medium per well were plated in 96-well plates and incubated for 24 h. Cells were then exposed to ParvD (0.1–25 µM) for 48 h. The CV assay was carried out according to previously described protocols [22,23,24]. Two or three independent experiments were performed, each comprising four replicate cultures. IC_50_ was calculated using GraphPad software 7.0.

### 2.5. Nuclear Morphology

ParvD-induced apoptosis in MDA-MB-231 cells was examined according to nuclear morphological changes using DAPI staining [25]. Cells were treated with different concentrations of ParvD (0; 0.1; 3 and 5 µM) for 8, 24, and 48 h. Later cells were fixed and stained with DAPI and analyzed by fluorescence microscopy. Cell image acquisition was performed using a wide field BX51 fluorescent Olympus microscope with a 40× objective. The number of apoptotic cells was determined according to changes in nuclear morphology, including shrinkage, condensation, margination, and chromatin fragmentation.

### 2.6. Cell DNA Content Analysis

MDA-MB-231 cells were cultured in 6-well plates for 24 h. Cells were then treated with different ParvD concentrations (0; 0.2 and 3 µM) with a 48-h incubation period. Subsequently, cells were harvested and with 5 mM EDTA in PBS and fixed with 80% ethanol. Following treatment with RNase A (20 µg/mL) and staining with PI (10 µg/mL) the cellular DNA content was analyzed by flow cytometry using a FACSCalibur flow cytometer (BD) [26,27]. Data acquisition and analysis were performed using CellQuest software (BD) and FlowJo (Tree Star, San Carlos, CA, USA), respectively.

### 2.7. Chemotaxis and Chemoinvasion

The chemotactic migration of MDA-MB-231 cells was assessed in 24-well plates which contained transwell inserts with a transparent PET membrane with 8 µm sized pores (BD Falcon, Bedford, MA, USA), according to Florido et al. [21]. Cells resuspended in the FBS-free medium were seeded on the top of the insert, and complete medium was placed in the lower chamber of the culture well. A ParvD concentration of 1 µM was added in both chambers and cells were incubated for 24 h. The evaluation of the chemotactic migration was performed following a previously described protocol [28]. The results were expressed as percentages of non-treated control cultures. The chemoinvasion assay was executed as described above for the chemotaxis assay, but coating the transwell inserts membrane with Matrigel diluted 30 times in serum-free medium. Three independent experiments were performed.

### 2.8. Cell Detachment Assay

To evaluate cell adhesion, MDA-MB-231 cells were cultured in 24-well plates for 24 h, to achieve a confluence of approximately 30%. Cells were then incubated with several ParvD concentrations (0, 1 and 2 µM) for a period of 24 h. The EDTA-induced cell detachment assay was performed following previously described protocols [28,29].

## 3. Results

### 3.1. P. ecklonii Extracts Preparation and HPLC-DAD Quantification

A multiple extraction study of *P. ecklonii,* which included aqueous, supercritical CO_2_ and acetonic extracts, was performed to select the method to isolate ParvD in highest amount. The extraction techniques involved were decoction, infusion, microwave, supercritical fluid, and ultrasound-assisted extractions. The quantification of ParvD on the different extracts was performed by HPLC-DAD, which indicated that the acetonic extracts have a higher ParvD concentration, when compared with the aqueous extracts; this was expected considering the chemical structure of ParvD. Acetonic extracts obtained by maceration or ultrasounds achieved ParvD concentrations of 136.8 and 166.1 µg/mg, respectively (Table 1).

The remaining extractions using water and supercritical CO_2_, independently of the extraction methods used (supercritical fluid extraction, decoction, infusion, microwave, and ultrasound extractions) only reached values of around 2.4 and 1.0 µg/mg, which are shown in Table 1. Considering the extraction efficiency, the acetonic ultrasound method was selected to isolate ParvD and spectroscopically confirmed ParvD was subsequently used in biological studies.

### 3.2. ParvD Reduces the Viability of MDA-MB-231 Cells

**Figure 2:**
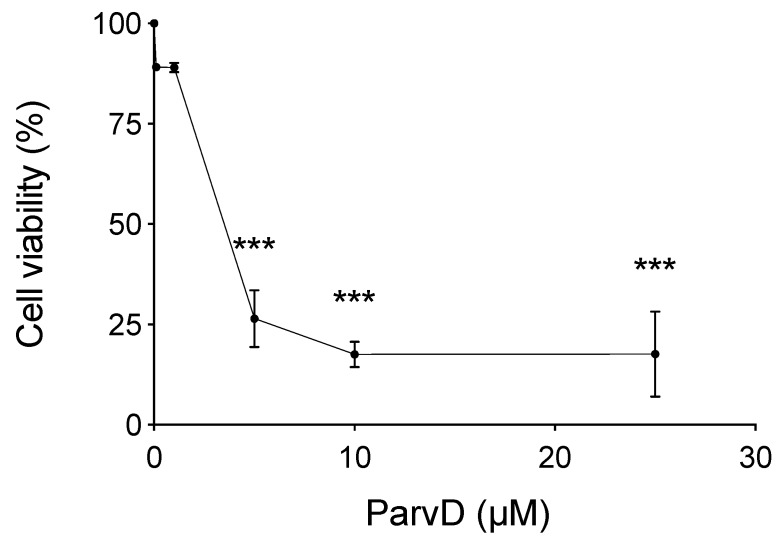
Exposure of cells to ParvD for 48 h reduces MDA-MB-231 cell viability measured by a crystal violet assay. Results are expressed as means ± SD (*n* = 2–3). *** *p* < 0.001 versus control (one-way ANOVA with Tukey’s multiple comparison test).

The cytotoxicity of ParvD was evaluated in MDA-MB-231 cells, a well-established in vitro model of TNBC. ParvD decreased cell viability in a concentration-dependent manner, following a 48 h incubation period. ParvD 0.1 and 1 µM demonstrated only a slight decrease in cell viability, with 89% of live cells by the end of the 48 h incubation period. Concentrations of ParvD above 5 µM showed a remarkable decrease in the cell viability, as it can be observed in Figure 2. The IC_50_ value was 2.48 µM.

### 3.3. ParvD Induces Apoptosis in MDA-MB-231 Cells

**Figure 3:**
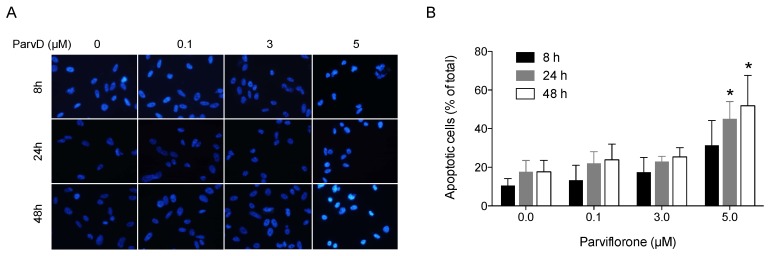
ParvD-mediated apoptosis in MDA-MB-231 cells was examined by nucleus morphological changes using DAPI. (**A**) Cells were treated with the indicated concentrations of ParvD for 8, 24, and 48 h, fixed stained with DAPI, analyzed under fluorescence microscopy, and the number of apoptotic cells was determined according to changes in nuclear morphology, including shrinkage, condensation, margination, and fragmentation of chromatin. Representative fields of cells are shown. (**B**) Summary results (means ± SD from at least 200 cells for each condition) show the percentage of apoptotic cells. * *p* < 0.05 (Student’s *t*-test, relative to untreated cells for the same incubation period).

Aiming at characterizing the reduction in cell viability provoked by ParvD, the induction of apoptosis was assessed by analyzing the cell nuclear morphology using DAPI staining. The results are depicted in Figure 3. The percentage of apoptotic cells increased with the concentration of ParvD and with the period of exposure. ParvD 5 µM showed a significant pro-apoptotic effect in MDA-MB-231 cells after periods of 24 and 48 h of incubation, increasing the % of apoptotic cells by 2.5- and 2.9-fold, respectively.

### 3.4. ParvD Increases the Sub-G1 Population

**Figure 4:**
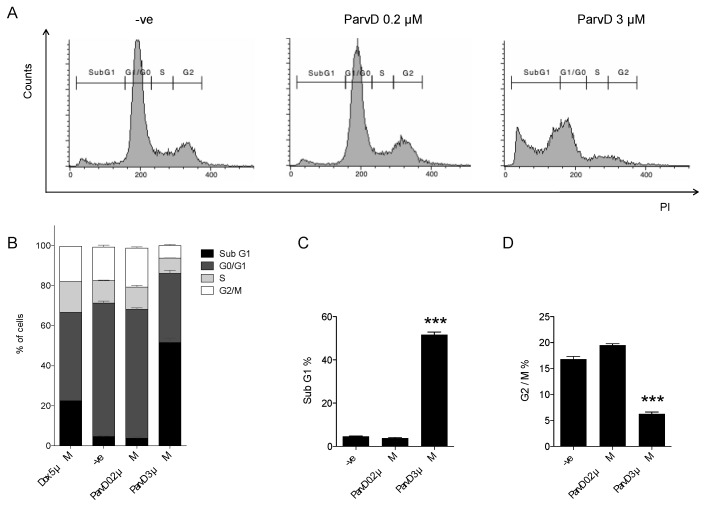
ParvD induces an increase in Sub-G_1_ and a reduction of G_2_/M populations. MDA-MB-231 cells were treated with the indicated concentrations of ParvD for 48 h. Dox 5 µM was used as a positive control. Cells were fixed, permeabilized, and stained with PI. (**A**) Cellular DNA content was analyzed by flow cytometry, representative histograms are shown. (**B**–**D**) Cell Sub-G_1_, G_0_/G_1_, S, and G_2_/M population summary results (Means ± SEM). *** *p* < 0.001 (Student’s *t*-test, relative to untreated cells).

The impact of ParvD on the cell cycle progression was evaluated by assessing the cellular DNA content by flow cytometry. As shown in Figure 4, while a low concentration of ParvD (0.2 µM) did not impact the cell cycle distribution, the exposure of cells for 48 h to a higher concentration (3 µM) led to a marked increase in the Sub-G_1_ phase. Indeed, a 13-fold increase in Sub-G_1_ cell population versus controls was observed. In parallel, the cell G_2_/M population suffered a three-fold decrease when compared with control cells. Cells treated with ParvD 3 µM show a two-fold higher percentage of Sub-G_1_ phase and three-fold lower percentage of G_2_/M phase when compared with cells treated with doxorubicin (Dox) 5 µM, a well-established anticancer drug used in breast cancer treatment.

### 3.5. ParvD Reduces Breast Cancer Cell Migration and Invasion

**Figure 5:**
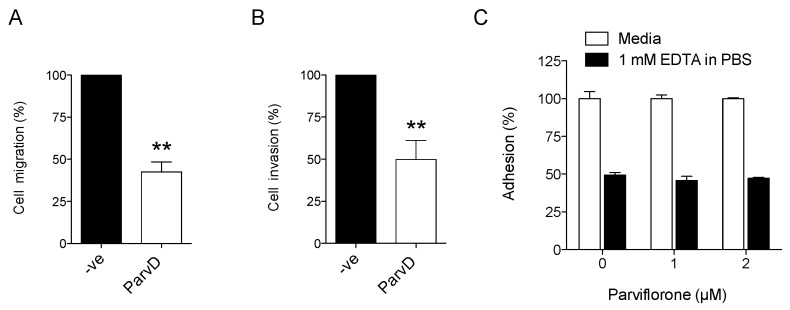
Impact of ParvD on cell migration, invasion, and detachment. Cell migration (**A**) and cell invasion (**B**) were evaluated using a transwell system. Results are expressed as mean values ± SD (*n* = 3) ** *p* < 0.01 (Student’s *t*-test, relative to untreated cells). Cell adhesion was assessed using an EDTA-induced cell detachment assay (**C**). Results are expressed as mean values ± SD (*n* = 2).

Given the importance of cell migration and cell invasion for cancer progression, especially for the formation of metastases, the effects of ParvD at this level was also assessed. The results are shown in Figure 5. ParvD (1 µM, 24 h), decreased the chemotactic migration of MDA-MB-231 cells by 57% when compared with the non-treated control cells (Figure 5A). Regarding cell invasion, a decrease of 50% was observed in cells treated with ParvD (Figure 5B).

As ParvD treatment influenced cell migration, the impact of this compound on cell adhesion/detachment was also determined. This was assessed using an EDTA-induced cell detachment assay, using experimental conditions that lead to ~50% cell detachment in cells not treated with ParvD. As shown in Figure 5C, ParvD (0–2 µM) did not affect cell detachment.

## 4. Discussion

Plants constitute a rich source of bioactive natural compounds with potential therapeutic effects for a wide number of diseases, including cancer. The selection of the procedure to extract bioactive compounds from their plants of origin is a critical step to obtain enough amounts of pure compounds that allow biological evaluation. In this work, the extraction method using acetone and an ultrasound-assisted technique is the one that results in the higher quantity of isolated ParvD. The acetone extraction results are in agreement with previous studies, where other bioactive abietane diterpenoids from *Plectranthus* spp. were isolated [9]. The highest efficiency of this extraction method is likely because of a more effective rupture of the plant cell wall, enabling the leaching of the ParvD into the acetone solvent [30].

Although ParvD is scarcely studied, previous reports have demonstrated a cytotoxic effect of ParvD in different cancer cell lines. Burmistrova et al. showed that ParvD had a remarkable cytotoxic effect in different human cancer cell lines. The leukemia cell lines HL-60, U-937, MOLT-3, and K-562 were especially susceptible to this compound, with IC_50_ values ranging from 0.35 to 1.2 µM after a 72-h period of incubation [7]. In another study, Silva et al. reported the cytotoxic effect of ParvD in melanoma cell lines B16V5 and A375 with IC_50_ values of 6.86 µM and 6.24 µM, following a 24 h period [12]. In comparison with the only reported study so far using the non-metastatic breast cancer cell line MCF7 [13] (IC_50_ value of 35.10 µM), our results demonstrate that ParvD is highly cytotoxic for MDA-MB-231 cells, with an IC_50_ value of 2.48 µM. This is an encouraging finding, since MDA-MB-231 cells exhibit a high level of resistance to an array of chemotherapeutic drugs [31]. Other diterpenoids previously isolated from distinct plant sources were also studied in MDA-MB-231 cells. The reported IC_50_ values were higher than those obtained in the present work [32,33,34].

Given the pronounced cytotoxic effect observed, we explored whether ParvD promoted apoptosis. Our data shows that ParvD increased the % of nuclei with morphological alterations characteristic of apoptotic cells. In line with this observation, an increase in the sub-G1 population was also found. Interestingly, the increase in the Sub-G_1_ population was more notorious for ParvD 3 µM than for Dox 5 µM, a chemotherapy drug frequently used in TBNC treatment modalities [35,36]. The pro-apoptotic effect described here for MDA-MB-231 cells was previously reported in other cancer cell lines. Burmistrova et al. reported an apoptotic effect of ParvD in the human myeloid leukemia cell lines HL-60 and U-937, demonstrated by the condensation and fragmentation of chromatin as well as by the increase in sub-G_1_ cell populations [7]. Similarly, Silva et al. reported an increase in Sub-G_1_ phase in the melanoma cell lines B16V5 and A375 [12]. Gelomulide K, another abietane diterpene, also induced apoptotic cell death in MDA-MB-231 cells, but a much higher concentration was needed to achieve a significant effect in comparison with ParvD (50 µM vs. 5 µM), after a 24 h period of incubation [33]. Although the mechanism by which ParvD induces apoptosis is not fully understood, a previous study [7] suggests that it might involve loss of mitochondrial membrane potential, extracellular signal-regulated kinases (ERK) 1/2 signaling, and reactive oxygen species accumulation.

The mortality associated with breast cancer is mostly due to the metastatic behavior of breast cancer cells [37]. Therefore, we studied the effects of ParvD on cell adhesion, cell migration, and cell invasion, which are determinant features for the formation of metastases. This is the first study addressing the impact of ParvD in these cellular processes. While ParvD had no effect in the adhesion/detachment of MDA-MB-231 cells, it led to a clear reduction in chemotactic cell migration and in cell invasion. Chemotaxis is a single-cell and directed type of cell migration, essential for tumor dissemination during progression and metastasis, including in the case of breast cancer [24,38]. The observed impact of ParvD in cell migration and cell invasion suggests that this compound might be useful in managing cancer dissemination and growth [38].

In summary, ParvD was isolated using a preliminary comparative extraction study, which allowed selecting an efficient ultrasound-assisted extraction. ParvD has shown anti-migration and anti-invasion properties and, at higher concentrations, cytotoxic and pro-apoptotic effects in an in vitro model of TNBC. Despite these encouraging results, ParvD is not selective toward cancer cells [7,12], as also happens with anticancer drugs currently in clinical use for TNBC [39,40]. A previous study using human normal-like fibroblasts (Detroit 551) determined an IC_50_ value of 9 µM for ParvD [12]. Therefore, future studies should be taken to develop adequate delivery systems that allow the targeting of this compound specifically to breast cancer cells. Such approaches were proposed by Silva et al. (2016) and Santos-Rebelo et al. (2018) using hybrid nanoparticles to target ParvD to melanoma and pancreatic cancer cells [12,13].

## 5. Conclusions

This is the first report describing the pro-apoptotic properties of ParvD on TNBC cells. Additionally, for the first time the anti-migratory effects of this compound were demonstrated, unraveling potential anti-metastatic properties of this natural compound. Although ParvD has shown encouraging effects in the breast cancer cell model used, further efforts must be undertaken to enhance the selectivity of this compound toward cancer cells, limiting undesirable side effects in non-cancer cells. Additionally, the dissection of the molecular mechanisms underlying the observed effects, as well as in vivo experiments will contribute to the understanding of the therapeutic relevance of this compound. 

## Figures and Tables

**Table 1:** Amount of parvifloron D in 1 mg of *P. ecklonii*.

Extraction Method	Parvifloron D (µg/mg)
Acetone Maceration	136.8 ^1^
Acetone Ultrasound	166.1 ^1^
Supercritical fluid extraction	2.2 ^1^
Decoction	2.4 ^1^
Infusion	1.0 ^1^
Microwave	1.2 ^1^
Ultrasound	1.2 ^1^

^1^ Amount of ParvD quantified by HPLC-DAD.

## Supplementary Materials

The following are available online at https://www.mdpi.com/2218-273X/10/1/158/s1. Figure S1: ^1^H-NMR spectrum of Parvifloron D (400 MHz, CDCl3). The datasets used and/or analyzed during the current study are available from the corresponding author on reasonable request.
