# Supplementary material for: Anti-Migratory and Pro-Apoptotic Properties of Parvifloron D on Triple-Negative Breast Cancer Cells
Source: Biomolecules. 2020 Jan 19;10(1):158. doi: 10.3390/biom10010158 (PMC7023143; doi:10.3390/biom10010158)
Supplement: Supplementary file 1 [file biomolecules-10-00158-s001.pdf]

Anti-migratory and pro-apoptotic properties of Parvifloron D on triple-negative breast cancer cells

Nuno Saraiva[1], João G. Costa[1,2], Catarina Reis[1,2], Nuno Almeida[1], Patrícia Rijo[1,2] and Ana Sofia Fernandes[1,*]

1 CBIOS, Universidade Lusófona Research Center for Biosciences & Health Technologies, Campo Grande 376, 1749-024 Lisboa, Portugal

2 Research Institute for Medicines (iMed.ULisboa), Faculty of Pharmacy, Universidade de Lisboa, Av. Professor Gama Pinto, 1649-003 Lisboa, Portugal

* Correspondence: ana.fernandes@lusofona.pt

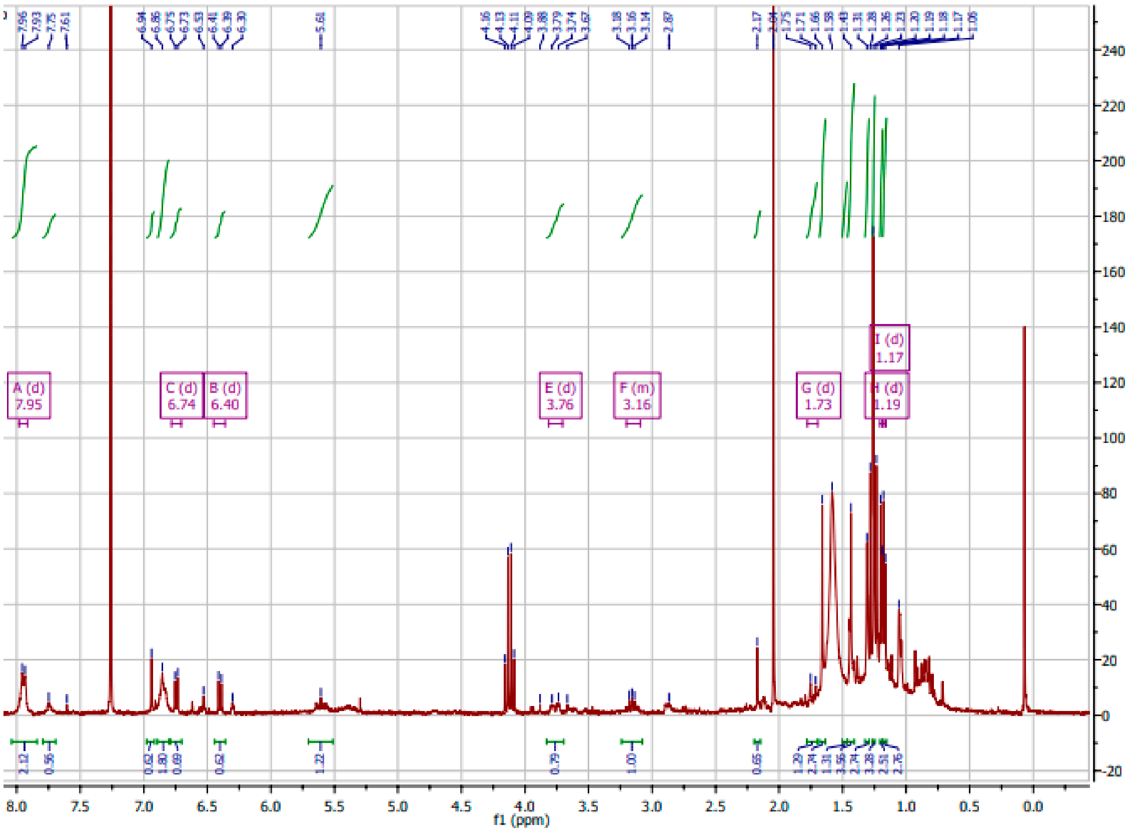

Figure S1. ^{1}H-NMR spectrum of Parvifloron D (400 MHz, $CDCl_3$).
